# Endoplasmic reticulum aminopeptidase 2 involvement in metastasis of oral cavity squamous cell carcinoma discovered by proteome profiling of primary cancer cells

**DOI:** 10.18632/oncotarget.18680

**Published:** 2017-06-27

**Authors:** I-Chun Kuo, Huang-Kai Kao, Yenlin Huang, Chun-I Wang, Jui-Shan Yi, Ying Liang, Chun-Ta Liao, Tzu-Chen Yen, Chih-Ching Wu, Kai-Ping Chang

**Affiliations:** ^1^ Department of Otolaryngology - Head & Neck Surgery, Chang Gung Memorial Hospital, Kwei-Shan, Tao-Yuan, Taiwan; ^2^ Department of Plastic & Reconstructive Surgery, Chang Gung Memorial Hospital, Kwei-Shan, Tao-Yuan, Taiwan; ^3^ College of Medicine, Chang Gung University, Kwei-Shan, Tao-Yuan, Taiwan; ^4^ Departments of Pathology, Chang Gung Memorial Hospital, Kwei-Shan, Tao-Yuan, Taiwan; ^5^ Molecular Medicine Research Center, Chang Gung University, Kwei-Shan, Tao-Yuan, Taiwan; ^6^ Departments of Nuclear Medicine, Chang Gung Memorial Hospital, Kwei-Shan, Tao-Yuan, Taiwan; ^7^ Department of Medical Biotechnology and Laboratory Science, College of Medicine, Chang Gung University, Kwei-Shan, Tao-Yuan, Taiwan

**Keywords:** OSCC, proteome, metastasis, prognosis, ERAP2

## Abstract

Oral cavity squamous cell carcinoma (OSCC) is a leading cause of cancer-related deaths worldwide and associated with poor prognosis and mortality. Discovery of proteins that can improve OSCC treatment is needed. Using comparative proteome profiling of primary cells derived from OSCC and adjacent noncancerous epithelium, endoplasmic reticulum aminopeptidases 2 (ERAP2) has been identified as an OSCC-associated protein. Compared with the adjacent normal tissues, ERAP2 levels were determined to be significantly elevated in OSCC tissues using quantitative real-time PCR and immunohistochemistry. Importantly, overexpression of ERAP2 was associated with positive N stage, advanced overall stage, positive perineural invasion, and tumor depth (*P* = 0.041, 0.015, 0.010, and 0.032, respectively). The overall survival rates of patients without and with the ERAP2 overexpression were 71.9% and 56.0%, respectively (*P* = 0.029). Furthermore, knockdown of ERAP2 inhibited the migration and invasion abilities of OSCC cells. Our results collectively show that ERAP2 overexpression is associated with the cervical metastasis and poorer prognosis of OSCC.

## INTRODUCTION

Head and neck cancers are the sixth most common malignancy worldwide, and more than 90% are squamous cell carcinomas. Oral cavity squamous cell carcinoma (OSCC) accounts for the vast majority of all head and neck squamous cell carcinomas [1]. Smoking, alcohol use, smokeless tobacco products, betel quid, inflammation, and oncogenic viruses are the major risk factors for OSCC [2]. Treatment modalities are mainly based on T staging and include surgery and adjuvant therapy including chemotherapy and radiotherapy. Cervical lymph node metastasis plays a leading role in predicting prognosis in OSCC. Previous studies have focused on diagnostic and therapeutic strategies for OSCC treatment; however, the 5-year disease-free survival rate has not significantly improved in advanced disease [3]. The discovery of biomarkers or OSCC-associated proteins may help improve the detection and/or treatment of OSCC.

To identify a potential biomarker for OSCC, we have established primary cell cultures derived from OSCC and adjacent noncancerous epithelium [4]. In the present study, we performed proteome profiling of primary cells using SDS-PAGE coupled with liquid chromatography-tandem mass spectrometry (GeLC-MS/MS). With spectral counting-based protein quantification [5], we revealed that expression of endoplasmic reticulum aminopeptidases 2 (ERAP2) was elevated in OSCC cells compared to noncancerous cells. ERAP2 belongs to the zinc-metallopeptidases of the oxytocinase M1 subfamily [6] and is encoded by the ERAP2 gene located on chromosome 5q15. ERAP2 involves in mechanisms of peptide trimming in endoplasmic reticulum for presenting human leukocyte antigen (HLA) class I molecules, shedding several cytokine receptors, and regulating angiogenesis [7–11]. However, the understanding of ERAP2 roles in cancer cells, especially OSCC cells, remains limited.

Herein, the expression levels of ERAP2 in OSCC tissues were determined using immunohistochemical (IHC) analyses and quantitative real-time polymerase chain reaction (RT-PCR). The capabilities of viability, migration, and invasion were evaluated in ERAP2-knockdown OSCC cells. We demonstrated that ERAP2 is overexpressed in OSCC tissues and involves in promotion of migration ability of OSCC cells.

## RESULTS

### Proteome profiling of primary cultured OSCC cells

To identify proteins involved in OSCC development and/or progression, the primary cancerous and adjacent noncancerous epithelial cells from oral cavities of three OSCC patients have been established (Table 1). The proteome between primary OSCC and noncancerous cells has been comparatively profiled to discover OSCC-associated proteins. Because of low protein output from the two primary OSCC cultures (Table 1), protein extracts from the two cancerous cultures were pooled [4]. For proteome analysis, proteins were initially separated using SDS-PAGE and stained with Coomassie blue. The gels were sliced into 20 fractions. Each gel slice was divided in 2 for technical duplicates, digested individually with trypsin, and analyzed using LC-MS/MS. A spectral search was performed against the Swiss-Prot database using the Mascot algorithm, and the results were further analyzed using Scaffold software [12]. Based on cutoffs of peptide probability of ≥ 0.95 and protein probability of ≥ 0.95, we detected 3197 and 3235 proteins with ≥ 2 peptide hits in the protein extracts from adjacent noncancerous cells and OSCC cells, respectively (Supplementary Table 1). Among 3774 non-redundant proteins, 2658 (70.43%) were found in both primary cultured cells, whereas 539 (14.28%) and 577 (15.29%) proteins were uniquely detected in the protein extracts from adjacent noncancerous cells and OSCC cells, respectively (Supplementary Table 1).

**Table 1:** Characteristics of tissue samples used for primary cell culture

Patient^a^	Established cultured cells	Sex	Age (years)	Site	TNM stage	Cell differentiation
A	adjacent noncancerous	male	50	tongue	T2N0M0	moderate
B	cancerous	male	43	buccal mucosa	T2N0M0	moderate
C	cancerous	male	51	floor of mouth	T2N0M0	moderate

^a^ The primary culture of cancerous cells for the patient A was unsuccessful. The primary culture of adjacent noncancerous epithelial cells for patients B and C failed

### Proteome-based discovery of OSCC-associated proteins

Using spectral count label-free quantification, we found that the levels of 221 proteins were significantly dysregulated in the primary OSCC cells compared with the primary adjacent noncancerous cells (Supplementary Table 2). Among them, 130 and 91 proteins were elevated and decreased, respectively, in cancerous versus noncancerous cells (Supplementary Table 2). To efficiently isolate potential OSCC markers, we used the Oncomine 4.5 database to retrieve the mRNA expression levels for the 221 proteins in the oral cavity tumor and noncancerous tissues. Four data sets were used (Supplementary Table 3): one comparing HNSCC with control tissues [13], two for oral cavity carcinoma versus normal tissues [14, 15], and one for tongue squamous cell carcinoma versus normal tissues [16]. Compared with noncancerous tissues, gene expression for 19 of 130 elevated proteins were found to be increased in cancer tissues (*P* < 0.05 cutoff) in all the evaluated data sets (Supplementary Table 4), while mRNA levels for 12 of 91 proteins were determined to be decreased (Supplementary Table 4). ERAP2, one of the elevated proteins, was found to be elevated in all four OSCC-related datasets were selected for further evaluation. Although it was perhaps arbitrary to select candidates based on gene expression alone, the use of a transcriptome analysis to narrow the proteins to the candidate markers seemed appropriate here.

### Overexpression of ERAP2 in OSCC tissues

**Figure 1:**
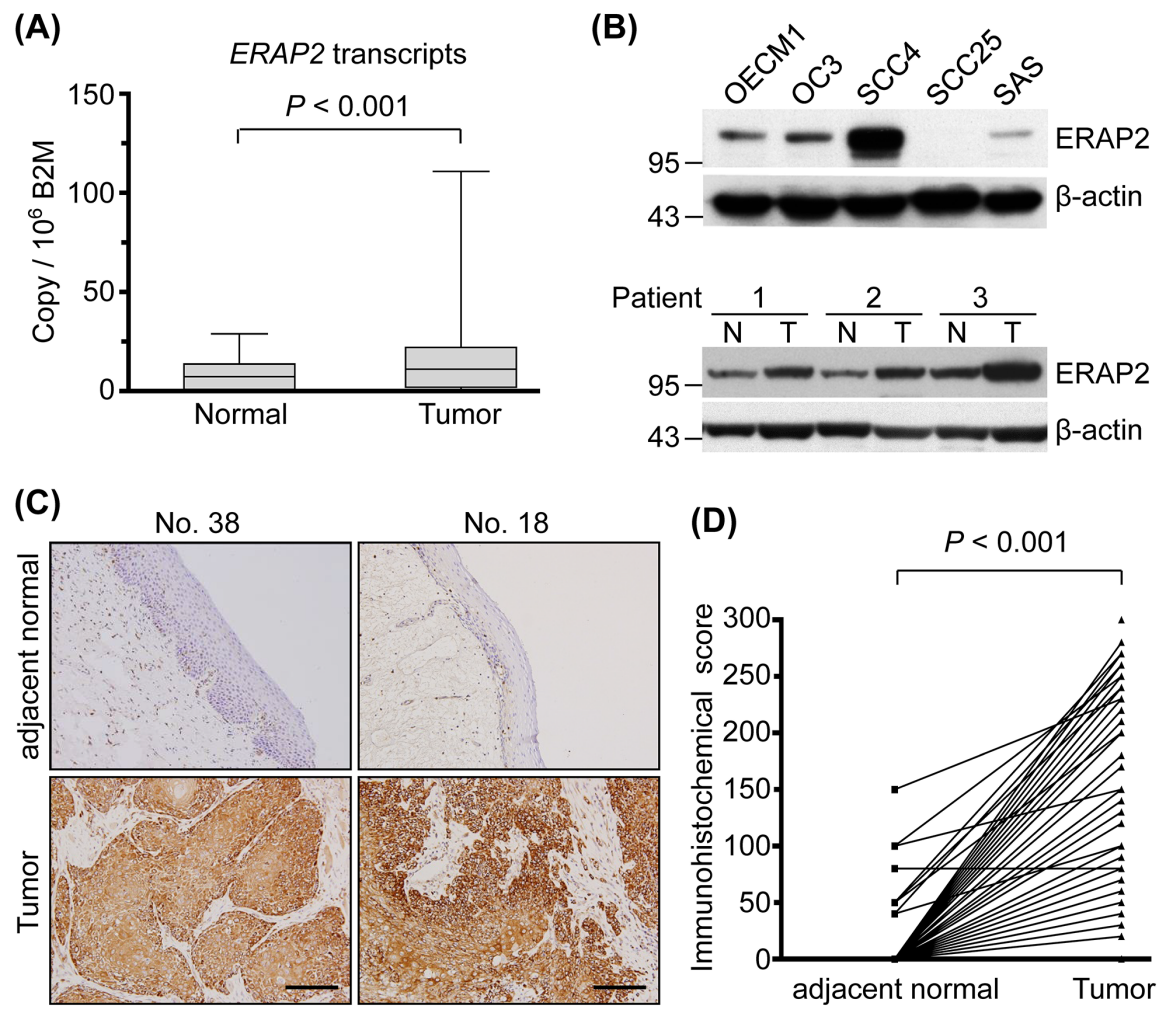
Overexpression of ERAP2 in OSCC tissues (**A**) Box and whisker plots showing ERAP2 mRNA transcript levels in the 40 paired normal and tumor tissues, as assessed by quantitative real-time RT-PCR. ERAP2 was highly overexpressed in OSCC tissues. (Box, the range of the middle 50% of ERAP2 level; line inside box, median; whiskers, minimal to maximal levels) **(B)** Western blot analysis of ERAP2 expression was shown in five OSCC cell lines and three paired tumor (T) and noncancerous (N) tissues. The β-actin signal was used as a loading control. **(C)** Immunohistochemical staining of ERAP2 in pericancerous adjacent normal epithelia (adjacent normal), and tumor tissues from two representative cases (scale bar = 100 μm). Expression patterns (brown staining) of ERAP2 expression indicate that this protein is localized in the tumor cell cytoplasm and membrane. **(D)** Statistical analysis for immunohistochemical scores of ERAP2 in 157 paired samples revealed higher ERAP2 expression levels in tumor cells than non-tumor normal epithelia (157.1 ± 79.04 *vs*. 5.0 ± 20.73, *P* < 0.001). The *P* value is obtained with paired *t* test.

To determine expression of ERAP2 in OSCC, ERAP2 transcripts were detected with quantitative RT-PCR in 40 paired OSCC and adjacent normal tissues. As shown in Figure 1A, the levels of ERAP2 transcripts in OSCC tissues were significantly elevated compared to that in the adjacent normal tissues (*P* < 0.001). The levels of ERAP2 mRNA between paired OSCC and normal tissues are also presented in a paired manner (Supplementary Figure 1A). The ERAP2 expression was further examined in OSCC and non-OSCC cell lines with immunoblotting. Compared to the noncancerous cells S-G and 293T, the ERAP2 can be detected in 4 OSCC cell lines, pancreatic cancer cell PANC1, ovarian cancer cell SKOV3, and kidney cancer cell 786-O (Figure 1B, upper panel and Supplementary Figure 1B). Furthermore, protein level of ERAP2 is elevated in tumor tissues compared to their noncancerous counterparts (Figure 1B, lower panel). To understand which cell type involved in overexpression of ERAP2 in OSCC tissues, we performed immunohistochemical staining of the tissue sections. As shown in Figure 1C, ERAP2 was highly expressed in the cytoplasm of tumor cells but was minimally detectable in the infiltrating lymphocytes and adjacent mesenchymal cells. Moreover, paired adjacent normal epithelium samples demonstrated lower or no ERAP2 expression (Figure 1C). Statistical analysis of the 132 paired samples available from these 157 patients demonstrated that ERAP2 expression was significantly higher in tumor cells versus normal epithelial cells (157.1 ± 79.04 *vs.* 5.0 ± 20.73, respectively; *P* < 0.001; Figure 1D).

### Association of ERAP2 expression with various clinicopathological manifestations

Next, we evaluated the relationships between increased ERAP2 expression and various clinicopathological characteristics in patients with OSCC (Table 2). Elevated ERAP2 expression was significantly associated with higher pN status, advanced overall stage, positive perineural invasion, and tumor depth (*P* = 0.041, 0.015, 0.010, and 0.032, respectively; Table 2 ). However, we observed no association between ERAP2 overexpression in OSCC tumors and patient age, sex, pT status, extracapsular spread, or differentiation.

**Table 2:** The clinicopathological characteristics related to the expression of ERAP2 in 157 samples of OSCCs

Characteristics	Case number	IHC score (Mean ± SD)	*P* value
Sex			
Male	137	160.3 ± 78.7	0.158
Female	20	134.3 ± 79.4	
Age (year)			
< 49.8	78	147.9 ± 80.3	0.084
≥ 49.8	79	166.0 ± 77.1	
T classification			
T1-T2	83	148.1 ± 84.7	0.080
T3-T4	74	167.0 ± 71.4	
N classification			
N = 0	101	147.8 ± 78.7	0.041^†^
N > 0	56	173.7 ± 77.5	
Overall stage			
I-II	59	139.8 ± 82.0	0.015^†^
III-IV	98	167.4 ± 75.7	
Extracapsular spread			
No	129	153.2 ± 80.1	0.132
Yes	28	177.5 ± 71.3	
Differentiation			
W-D + M-D	144	157.7 ± 77.7	0.969
P-D	13	150.0 ± 95.3	
Perineural invasion			
negative	112	146.6 ± 79.7	0.010^†^
positive	44	182.7 ± 72.4	
Tumor depth			
< 8mm (median)	84	145.8 ± 82.6	0.032^†^
≥ 8mm	72	169.5 ± 73.4	

^†^statistically significant. IHC, immunohistochemical; SD, standard deviation; M-D, moderately differentiated; P-D, poorly differentiated; W-D, well-differentiated

### Association of ERAP2 expression with overall survival (OS) and disease-free survival (DFS)

**Figure 2:**
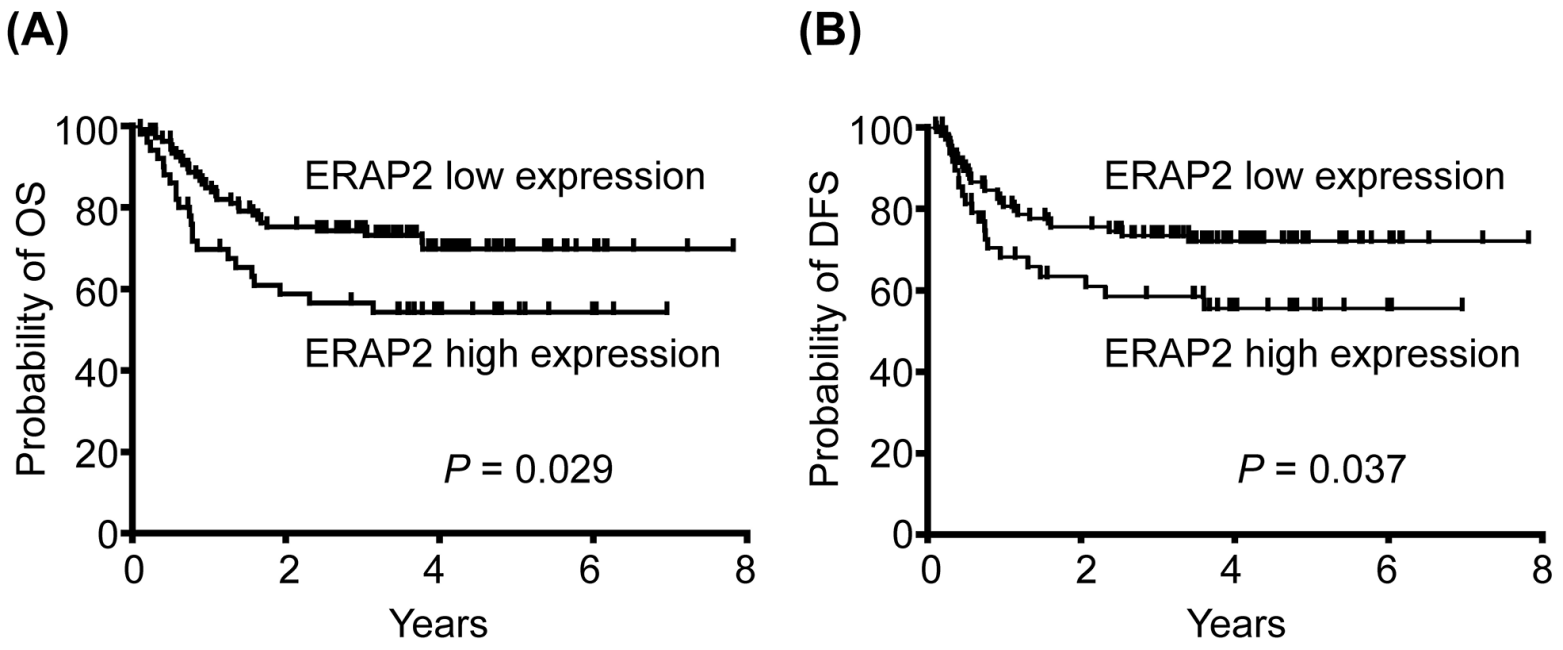
Association of high ERAP2 expression with poorer prognosis of patient survival (**A**) Kaplan-Meier plot for overall survival (OS) indicated that the 5-year OS rates for patient subgroups stratified by ERAP2 expression were 71. 9% *vs.* 56.0%, respectively (*P* = 0.029). **(B)** Five-year disease-free survival (DFS) rates for patients stratified by ERAP2 expression were 73.8% *vs.* 60.0%, respectively (*P* = 0.037).

Based on expression data obtained from IHC, patients were stratified into 2 groups (high *vs*. low expression using 150 out of 300 as the cut-off value), and we evaluated the possible association of ERAP2 expression with OS. Our survival analysis revealed that the 5-year OS rates for patients stratified into high and low ERAP2 expression subgroups were 71.9% and 56.0%, respectively. These differences in OS were significant in a log-rank test (*P* = 0.029; Figure 2A). Moreover, the 5-year DFS rates for patients stratified based on high or low ERAP2 expression were also significantly different in the log-rank test (73.8% and 60.0%, respectively; *P* = 0.037) (Figure 2B).

### ERAP2 involvement in the viability, migration, and invasion of OSCC cells

**Figure 3:**
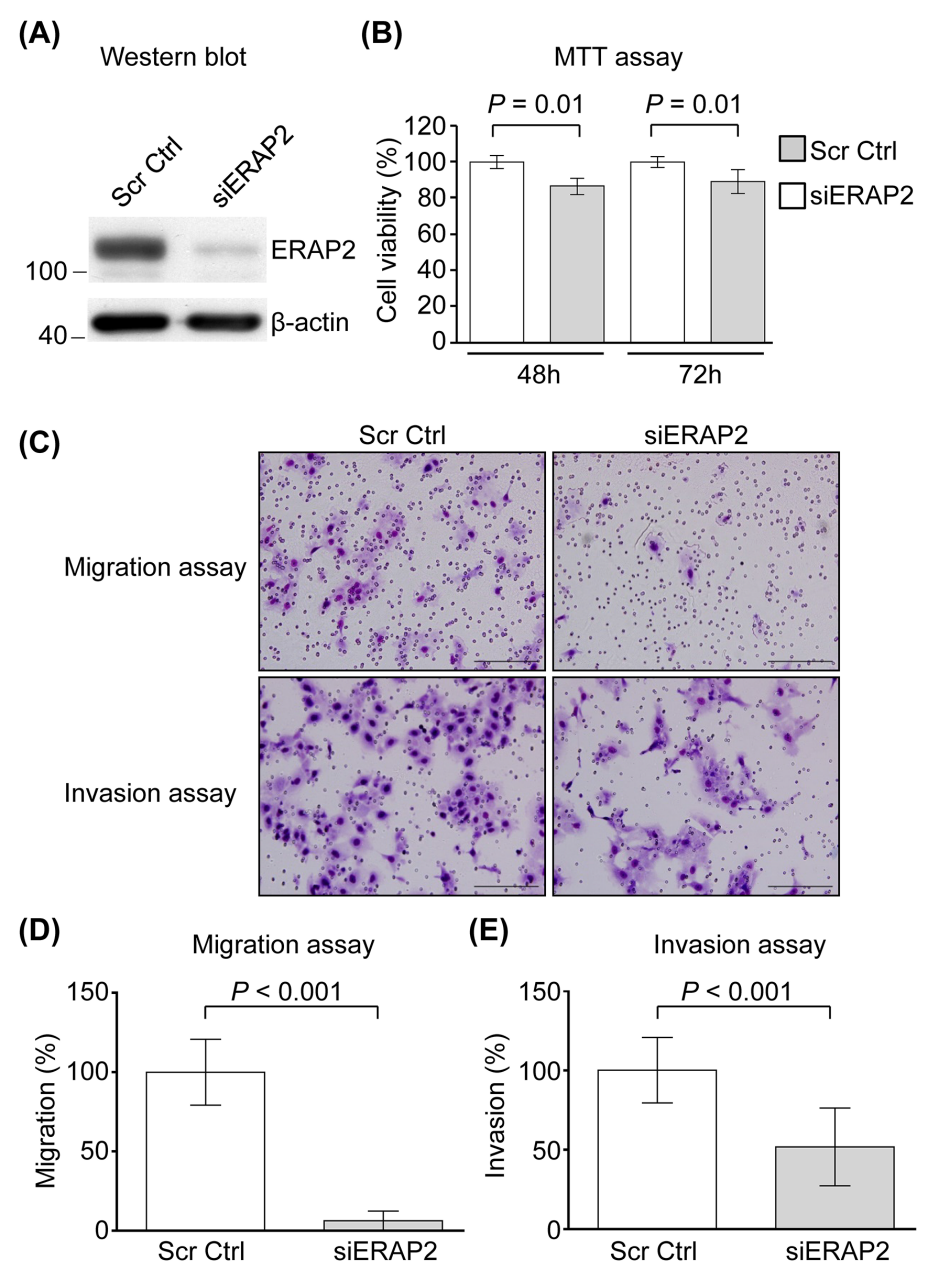
Involvement of ERAP2 in viability, migration, and invasion of OSCC cells (**A**) SCC4 cells were transfected with control siRNA (Scr Ctrl) and ERAP2 siRNA (siERAP2), respectively. After 48 h, protein extracts (20 μg) were prepared and ERAP2 was detected with Western blot. **(B)** Quantitative analysis of the MTT assay. Data are presented as mean values obtained from three independent experiments. Error bars indicated the standard deviation. **(C)** Representative microphotographs of filters obtained from the 16 h transwell migration **(D)** and 24 h invasion **(E)** assays. Original magnification: 200×. The migration (D) and invasion (E) assays were performed using SCC4 cells in which ERAP2 was eliminated.

To evaluate the biological significance of ERAP2 overexpression in OSCC progression, we applied siRNA approach to suppress the expression of ERAP2 in OSCC cells. Based on the finding that the expression level of ERAP2 is the most abundant in SCC4 cells among the OSCC cell lines tested (Figure 1B, upper panel), the SCC4 cell line was selected for knockdown of ERAP2. The effects of RNAi were firstly determined using Western blot in the SCC4 cells transfected with either *ERAP2*-specific RNAi or scrambled sequence control RNAi. As shown in Figure 3A, the expression of endogenous ERAP2 was significantly reduced in siERAP2-transfected cells compared with cells transfected with control RNAi. Control and siERAP2-transfected cells were further analyzed for cell viability, migration, and invasion*.* Cell viability was slightly decreased in siERAP2-transfected cells compared to the control RNAi-transfected cells (*P* = 0.01; Figure 3B). And the effect was marginal (∼10%). Furthermore, migration and invasion ability of ERAP2-knockdown cells were severely impaired, as compared with the control cells (Figure 3C and 3D). The migration and invasion capabilities were reduced to 17% and 52%, respectively, in ERAP2-knockdown SCC4 cells. Collectively, these findings indicated that ERAP2 is involved in cell migration and survival during OSCC tumorigenesis.

## DISCUSSION

OSCC is a leading cause of cancer-related deaths worldwide and frequently associated with poor prognosis and mortality. The discovery of biomarkers may help improve the detection and/or treatment of OSCC [4, 17]. To identify OSCC-associated proteins, we established primary cell cultures from paired OSCC and adjacent noncancerous tissues from three OSCC patients (Table 1). In the current study, we performed proteome profiling for established primary cells using GeLC-MS/MS and identified that ERAP2 could play a vital role in the metastasis of OSCC tumors, suggesting that proteome analysis of primary oral epithelial cells is a feasible strategy for OSCC biomarker identification, and that ERAP2 is a potentially useful tissue marker for the prognosis of OSCC.

The differential expression of ERAP1 and ERAP2 has been associated with several cancer types, including thyroid, lung, breast, colon, and endometrial cancers [18–21]. In this study, for the first time, we reveal that ERAP2 is upregulated in OSCC tissues (Figure 1) and its overexpression correlates with poor survival of OSCC patients (Figure 2). The suppression of ERAP expression results in the aberrant levels of surface HLA class I molecules and consequently leads to alteration of ability in peptide trimming of tumor cells [8, 22, 23]. These findings suggest the pivotal role of ERAP in presenting antigenic epitopes to T lymphocytes. The inaccuracy of antigen processing impairs immune surveillance and contributes to immune evasion of tumor cells. Indeed, the activities of ERAP for immune escape of cancer cells have been revealed [24, 25], suggesting the poor survival of OSCC patients with elevated level of ERAP2 may result from the ERAP2-associated immune evasion.

The overexpression of ERAP2 has been demonstrated to be correlated with the poorer clinicopathological characteristics, including cervical lymph node metastasis, advanced overall stage, positive perineural invasion, and deeper tumor depth (Table 2), indicating that elevated ERAP2 levels contributed to poorer prognosis of OSCC patients. Furthermore, the inhibition of endogenous ERAP2 expression can bring about the decreased capabilities of migration and invasion in the OSCC cells (Figure 3). Previously, ERAP2 level also has been reported to be a molecular marker that could predict cervical lymph node metastasis in papillary thyroid microcarcinoma [20], suggesting that ERAP2-induced promotion of cell migration may lead to poorer prognosis of OSCC.

The mainstream therapeutic modalities for OSCC nowadays are surgery with or without adjuvant treatments including radiotherapy and chemotherapy, but there has been no major breakthrough in patient survival over the recent decade. For the management of OSCC tumors, gene therapy has been recently considered because of compelling evidence and given its importance in cancer therapy. Based on our findings, ERAP2 may have the potentials to be a target for pharmacological or genetic modification in OSCC tumors. However, additional investigation is required to clarify the modulating mechanism of ERAP2 in OSCC.

## MATERIALS AND METHODS

### Patient characteristics and clinical specimens

Tissues used for the establishment of primary cell cultures were collected from 3 OSCC patients diagnosed at the Chang Gung Memorial Hospital (Tao-Yuan, Taiwan) in 2010 (Table 1). Tumor specimens for IHC analyses were obtained from 157 consecutively enrolled patients (137 men and 20 women) with OSCC diagnosed at the Chang Gung Memorial Hospital (Tao-Yuan, Taiwan) between August 2002 and November 2007. Patient age at diagnosis ranged from 22.0 to 85.0 y (mean, 51.7 ± 12.3). The associated oral cavity subsites were the buccal mucosa (61 patients), gum (20), hard palate (5), lip (4), floor of the mouth (7), and tongue (60). Patients with at least one of the following conditions were considered ineligible: an unresectable tumor, concomitant other primary cancer (synchronous or metachronous), recurrent disease, distant metastasis at presentation, prior history of any malignancy, treatment with neoadjuvant therapy, medical contraindication for surgery. All patients provided informed consent prior to study participation; the Institutional Review Board of Chang Gung Memorial Hospital approved the study. Patients underwent standard preoperative work-ups according to institutional guidelines, including detailed medical history, complete physical examination, head and neck computed tomography or magnetic resonance imaging scans, chest radiographs, bone scan, and abdominal ultrasound. Primary tumors were excised with adequate margins using intraoperative frozen section control. Plastic surgeons immediately reconstructed any surgical defects using a free flap or local flap, if necessary. Following surgical treatment, pathological TNM classification of all tumors was established according to the American Joint Committee on Cancer Staging Manual (2010). After discharge, all patients had regular follow-up visits every 2-3 months for the first year, every 3-4 months for the second year, and every 6 months thereafter [26–28].

### Cell lines

OSCC cell line OECM1 cells were cultured in RPMI 1640 medium (RPMI). SCC4 cells were grown in Dulbecco’s modified Eagle’s medium (DMEM). SCC25 cells were maintained in RPMI and DMEM/F12 (1:1) media. OC3 cells were cultivated in DMEM and KSFM (1:2) media. SAS cells were cultured in DMEM/F12 medium. Human gingival epithelial cell S-G, breast cancer cell MCF7, embryonic kidney cell 293T and pancreatic cancer cell PANC1 were grown in DMEM. Human ovarian carcinoma cell SKOV3 was maintained in DMEM/F12 medium. Human renal cell adenocarcinoma cell 786-O was cultivated in RPMI. All media were purchased from Invitrogen (Carlsbad, CA, USA) and contained 10% FBS and antibiotics. All the cells were maintained at 37°C in a humidified atmosphere of 95% air and 5% CO_2_.

### Preparation of cell extracts and digestion of protein mixtures for proteome analysis

Cells were lysed in 0.1% RapiGest^TM^ SF (Waters Corporation, Milford, MA, USA) on ice for 15 min. The protein mixtures were separated using SDS-PAGE and subjected to in-gel tryptic digestion as described in the Supplementary Materials.

### Peptide fractionation and LC-MS/MS analysis

The in-gel digested peptides were loaded across a trap column (Zorbax 300SB-C_18_, 0.3 × 5 mm, Agilent Technologies, Wilmington, DE, USA) and separated on a resolving 10-cm analytical C_18_ column (inner diameter, 75 μm) with a 15-μm tip (New Objective, Woburn, MA, USA). The peptides were eluted as described in the Supplementary Materials. The reversed-phase LC apparatus was online and coupled to a mass spectrometer. Peptides were analyzed with an LTQ-Orbitrap Discovery (Thermo Fisher Scientific, Taipei, Taiwan) as described in the Supplementary Materials.

### Database search for protein identification and quantitation

For a database search, the obtained MS/MS spectra were analyzed using the Mascot algorithm (version 2.1, Matrix Science, MA, USA) against the Swiss-Prot human sequence database (released Apr 16, 2014, selected for *Homo sapiens*, 20265 entries) from the European Bioinformatics Institute. After a Mascot search, the obtained files were processed using Scaffold software (version 3.6.5; Proteome Software, OR, USA). Detailed parameters regarding protein identification are described in the Supplementary Materials.

To comparatively quantify proteins, we performed a label-free comparison between cancerous and noncancerous cells using a spectral counting method [12]. The numbers of spectra assigned to each protein were exported from the Scaffold software in an MS Excel format. Normalized spectral count (SC) for each protein was obtained by dividing the SC for a given protein by the total SC obtained from the experiment. Fold change was determined by dividing the average normalized SCs for the cancerous group by that of the noncancerous group. We failed to identify all proteins in all experiments; unidentified proteins or missing values in a particular sample were assigned an SC equal to one to avoid dividing by zero and to prevent overestimation of fold changes. After log_2_ transformation of protein fold changes, proteins with log_2_ ratios below the mean of all ratios minus two standard deviations (SD) of all ratios (-3.438) were considered to be underexpressed, while those above the mean plus two SD (3.488) were considered to be overexpressed.

### RNA extraction and quantitative RT-PCR

Forty paired OSCC tumor and pericancerous normal tissues were homogenized in liquid nitrogen with a mortar and pestle and incubated with RNAzol B reagent (Tel-Test, Friendwood, TX). The RNA was further purified using an RNeasy cleanup kit (Qiagen, Valencia, CA, USA) according to the manufacturer’s protocol. First-strand cDNA was synthesized from 5 μg of total RNA and then mixed with a reaction mixture consisting of commercially available primers (ERAP2 Hs01073631_m1 and normalization control B2M, Hs00984230_m1; Assay-on-Demand, Applied Biosystems, Foster City, CA, USA), RNase-free water, and TaqMan Universal PCR Master Mix. Quantitative RT-PCR was performed and analyzed using a 7900 HT Sequence Detection System and SDS version 2 (Applied Biosystems). All experiments were repeated in duplicate.

### Western blot analysis

Proteins were extracted from culture cells using an RIPA buffer (50 mM Tris pH 8, 0.0150 mM NaCl, 2 mM EDTA, 1% Triton X-100, 0.1% SDS, 0.2% Na-deoxylate, 1× protease cocktail [Sigma-Aldrich, St. Louis, MO, USA]); the concentrations were determined using a Pierce BCA protein assay kit (Thermo Fisher Scientific, Rockford, IL, USA). Samples were separated on 8% SDS gels, transferred to PVDF membranes (GE Healthcare Life Sciences, Buckinghamshire, UK), and probed using mouse monoclonal LRAP/ERAP2 antibody (MAB3830, R&D systems, Minneapolis, MN, USA) and mouse monoclonal β-actin antibody (MAB1501, Chemicon, Billerica, MA, USA). The β-actin signal was used as a loading control.

### Immunohistochemical staining

For immunohistochemistry, formalin-fixed and paraffin-embedded tissues were cut into 4-μm sections, deparaffinized, rehydrated, and prepared for antigen retrieval. Slides including consecutive sections were incubated with the appropriate antibodies: anti-LRAP/ERAP2 antibody (diluted 1:30, AF3830, R&D systems) at room temperature for 15 min. After incubation, the slides were washed 3 times with phosphate-buffered saline (PBS), incubated with a horseradish peroxidase (HRP) polymer antibody (Invitrogen) at room temperature for 10 min, and developed by adding 3,3’-diaminobenzidine tetrahydrochloride (DAB) reagent (Dako, Glostrup, Denmark), with chromogen and hematoxylin as a counterstain. Images of the stained slides were obtained using a ScanScope CT automated slide-scanning system (Aperio Technologies, Vista, CA, USA). ERAP2 expression was scored using a combined scoring method accounting for both staining intensity and percentage of stained cells as described in the Supplementary Materials [29–31]. All specimens were independently evaluated by our pathologists (Y Liang and YL Huang). Immunohistochemical scores exceeding 150 were classified as high ERAP2 expression.

### Knockdown of ERAP2 with RNA interference (RNAi)

SMARTpool small-interfering RNAs (siRNA) were purchased from Dharmacon (Lafayette, CO, USA). RNAi specifically targeting human *ERAP2* (No. L-005934-00-0005, Dharmacon) and a scrambled control RNAi (No. D-001810-10-05, Dharmacon) were purchased from Thermo Fisher Scientific. RNAi (at a final concentration, 400 nM) was mixed with Lipofectamine RNAiMAX^TM^ (Invitrogen) and Opti-MEM medium (Invitrogen) without serum, incubated for 20 min at room temperature, and added to SCC4 cells that were seeded at a density of 1 × 10^5^ cells per well in 6-well plates. After incubation for 6 h at 37°C, transfer fresh culture medium (DMEM/F12 medium containing 10% FBS) was added to each well. After transfection for 48 h, cells were harvested for analysis of cell migration and invasive capacity.

### Cell function assay

Cell viability was determined using a methylthia zoltetrazolium (MTT) assay (Bionovas Biotechnology, Toronto, Canada). Three independent experiments were performed in quadruplicate. The average value of the control experiment was taken as 100% viability and used to calculate the percentage of cell viability for each treatment. Cell migration was evaluated using a chemotaxis chamber (Corning, Lowell, MA, USA) with a polycarbonate membrane (8-μm pore size) placed between the 2 chambers. After 16 h incubation at 37°C, chambers were gently washed twice with PBS and fixed with methanol, followed by Giemsa staining. The cell invasion assay used BD biocoat matrigel invasion chambers (354480; BD Biosciences, Bedford, MA, USA). Transfected SCC4 cells (1 × 10^5^) that were in a 500-μL serum-free culture medium were applied to the upper chamber, and 750 μL of DMEM/F12 medium containing 10% FBS medium was added to the lower chamber. After 24 h incubation at 37°C, chambers were washed with PBS, fixed with methanol, and followed by Giemsa staining. Each migration and invasion assay was performed twice in 3 independent experiments. The details of these experiments were described in our previous reports [29–31].

### Statistical analysis

All statistical data are expressed as mean ± SD. The Wilcoxon signed-rank test was employed for comparison of the relative signal intensity for quantitative RT-PCR and immunohistochemical staining scores between paired tumor and pericancerous normal mucosa samples. Cell viability, growth, and migration experiments were compared using unpaired Student’s *t*-tests. All patients received follow-up evaluations at our outpatient clinic until October 2013 or death. A survival analysis was plotted using the Kaplan–Meier method; differences were evaluated using the log-rank test. Statistical analyses were performed using SAS software (version 9.1; SAS institute, Cary, NC, USA). All *P* values were 2-sided; significance was considered at a *P* value of <0.05.

## SUPPLEMENTARY MATERIALS FIGURE AND TABLES











## Abbreviation

DFS: disease-free survival
ERAP2: endoplasmic reticulum aminopeptidases 2
HLA: human leukocyte antigen
IHC: immunohistochemistry
MTT: methy-lthiazoltetrazolium
OS: overall survival
OSCC: oral cavity squamous cell carcinoma
PCR: polymerase chain reaction
SC: spectral count
