# Supplementary material for: Endoplasmic reticulum aminopeptidase 2 involvement in metastasis of oral cavity squamous cell carcinoma discovered by proteome profiling of primary cancer cells
Source: Oncotarget. 2017 Jun 27;8(37):61698–708. doi: 10.18632/oncotarget.18680 (PMC5617457; doi:10.18632/oncotarget.18680)
Supplement: Supplementary file 1 [file oncotarget-08-61698-s001.pdf]

Endoplasmic reticulum aminopeptidase 2 involvement in metastasis of oral cavity squamous cell carcinoma discovered by proteome profiling of primary cancer cells

SUPPLEMENTARY MATERIALS

Preparation of cell extracts and digestion of protein mixtures for proteome analysis

Cells were lysed with 0.1% RapiGest™ SF (Waters Corporation, Milford, MA, USA) on ice for 15 min. The cell lysate was collected and sonicated on ice, followed by centrifugation at 10000 × g for 25 min at 4°C. The resulting supernatant was used as the cell extract. Proteins immunoprecipitated from 3xFLAG NS1-expressing cells were separated by 10% SDS-PAGE and stained using a Colloidal Blue Staining kit (Thermo Fisher Scientific, NY, USA). The stained gel lanes were cut into 20 slices and subjected to in-gel tryptic digestion as described previously. Briefly, gel pieces were destained in 10% methanol (Mallinckrodt Baker, NJ, USA), dehydrated in acetonitrile (ACN; Mallinckrodt Baker) and dried using a SpeedVac. The gel pieces were treated with 25 mM NH_4HCO_3 containing 10 mM dithiothreitol (Biosynth AG, Switzerland) at 60°C for 30 min, followed by alkylation with 55 mM iodoacetamide (Amersham Biosciences, UK) at room temperature for 30 min. The proteins were then digested using sequencing-grade modified porcine trypsin (20 µg/mL; Promega, WI, USA) at 37°C for 16 h. Peptides were extracted with ACN and dried using a SpeedVac.

Peptide fractionation and LC-MS/MS analysis

Each peptide mixture was reconstituted in HPLC buffer A (0.1% formic acid; Sigma-Aldrich), loaded onto a trap column (Zorbax 300SB-C_{18}, 0.3 × 5 mm; Agilent Technologies, Taiwan) at a flow rate of 0.2 µL/min in HPLC buffer A, and separated on a resolving 10-cm analytical C_{18} column (inner diameter, 75 µm) with a 15-µm tip (New Objective, MA, USA). Using a flow rate of 0.25 µL/min across the analytical column, the peptides were eluted using a linear gradient of 0-10% HPLC buffer B (99.9% ACN containing 0.1% formic acid) for 3 min, 10-30% buffer B for 35 min, 30-35% buffer B for 4 min, 35-50% buffer B for 1 min, 50-95% buffer B for 1 min, and 95% buffer B for 8 min.

The LC apparatus was coupled online with a two-dimensional linear ion trap mass spectrometer (LTQ-Orbitrap Discovery, Thermo Fisher Scientific) managed using the Xcalibur 2.0 software package (Thermo Fisher Scientific). An electrospray voltage of 1.8 kV was applied. Intact peptides were detected by the Orbitrap at a resolution of 30000. The ion signal of $(Si(CH_3)_2O)_6H^+$ at *m/z* 445.120025 was used as an internal standard for mass lock. For MS analysis, we used a data-dependent acquisition mode that alternated between one MS scan and six MS/MS scans for the six most abundant precursor ions. For MS scans, the *m/z* scan range was set to 350-2000 Da. The *m/z* values selected for MS/MS scans were dynamically excluded for 3 min, and 5×10^4 ions were accumulated and resolved in the ion trap to generate MS/MS spectra. Both MS and MS/MS spectra were acquired using one microscan with maximum fill times of 1000 ms and 100 ms for MS and MS/MS analyses, respectively. Automatic gain control was applied to prevent overfilling of the ion trap.

Protein database searching for protein identification and quantitation

For database searching, the obtained MS/MS spectra were analyzed using the Mascot algorithm (version 2.1, Matrix Science, MA, USA) against the Swiss-Prot human sequence database (released Apr 16, 2014, selected for *Homo sapiens*, 20265 entries) of the European Bioinformatics Institute. The mass tolerances of the fragment and parent ions were set to 0.5 Da and 10 ppm, with trypsin as the digestion enzyme. Up to one missed cleavage was permitted, and searches were performed with the parameters of variable oxidation on methionine (+15.99 Da) and fixed carbamidomethylation on cysteine (+57 Da). A random sequence database was used to estimate false-positive rates for peptide matches.

After Mascot searching, the obtained files were processed using Scaffold software (version 3.6.5; Proteome Software, OR, USA), which includes the PeptideProphet program to assist in the assignment of peptide MS spectra and the ProteinProphet program for assigning/grouping peptides to a unique protein/protein family when they are shared among several isoforms. We used PeptideProphet and ProteinProphet probabilities ≥ 0.95 to ensure an overall false-discovery rate below 0.5%. Only proteins with two or more identified peptides were retained in this study.

To comparatively quantify proteins, we performed label-free comparison between the cancerous and noncancerous cells. The numbers of spectra assigned to each protein were exported from the Scaffold software in MS Excel format. The normalized spectral count (SC) of each protein was obtained by dividing the SC of a given

protein by the total SC in the experiment. The fold change was determined by dividing the average of normalized SCs for the cancerous group by that for the noncancerous group. We failed to identify all proteins in all experiments; unidentified proteins or missing values in a particular sample were assigned an SC of one to avoid dividing by zero and to prevent overestimation of fold changes.

IHC scoring

Expression of ERAP2 was scored using a combined scoring method that accounts for both the staining intensity and the percentage of stained cells. Strong, moderate, weak and negative staining intensities were scored as 3, 2, 1 and 0, respectively. For each of the intensity scores, the percentage of cells that stained at such level was estimated visually. The resulting combined score was calculated as the sum of the percentage of stained cells multiplied by the intensity scores. For example, a case with 20% weak staining, 30% moderate staining, and 50% strong staining would be assigned a score of 230 (20 × 1 + 30 × 2 + 50 × 3 = 230) out of a possible score of 300. The specimens were independently evaluated by two pathologists (Y Liang and YL Huang), without prior knowledge of their clinical data.

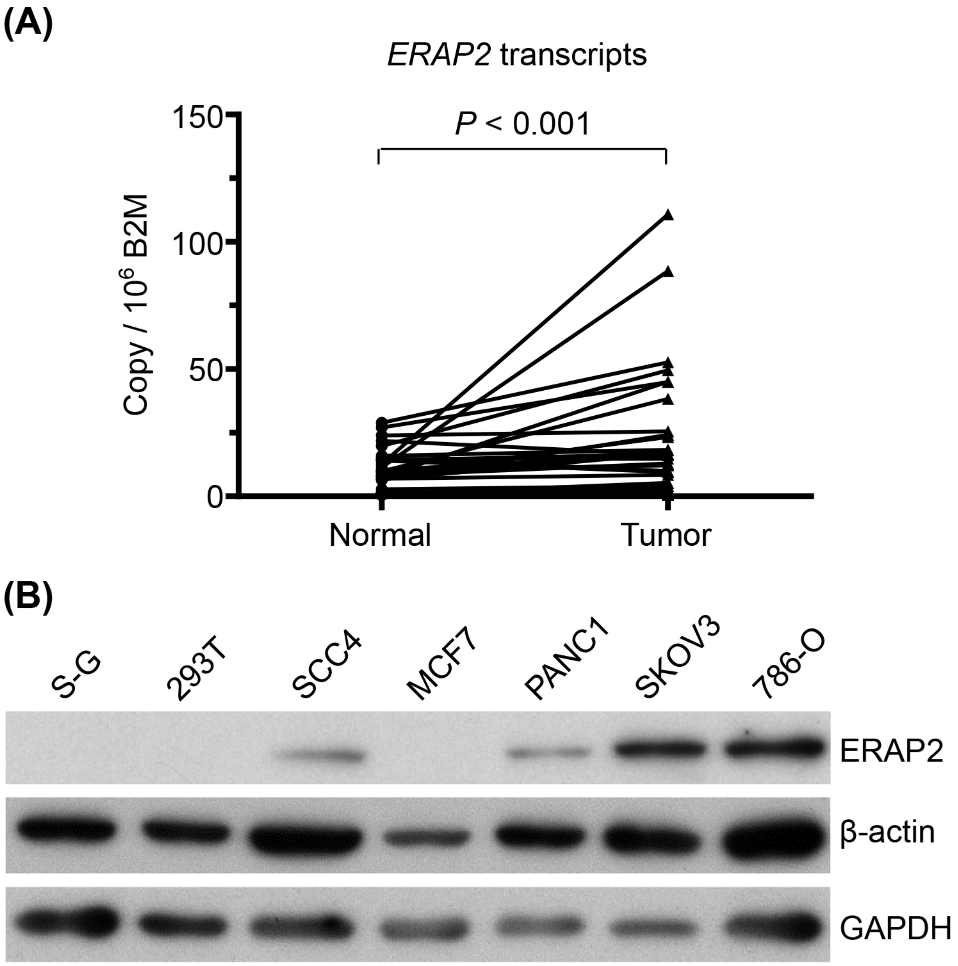

Supplementary Figure 1: ERAP2 expression in oral cancer tissues and cells. **(A)** Statistic alanalysis of ERAP2 mRNA transcript levels in the 40 paired normal and tumor tissues, a *p* value of less than 0.05 indicates significance based on the paired *t* test. **(B)** ERAP2 levels in human gingival epithelial cell S-G, embryonic kidney cell 293T, oral cancer cell SCC4, breast cancer cell MCF7, pancreatic cancer cell PANC1, ovarian cancer cell SKOV3,and kidney cancer cell 786-O were detected with Western blot. Protein samples (30 µgperlane) were separated by SDS-PAGE and detected with primary antibodies. β-actin and GAPDH were used as the loading control.

Supplementary Table 1: List of proteins identified in noncancerous and cancerous cells

See Supplementary File 1

Supplementary Table 2: List of proteins differentially expressed in primary cultured OSCC cells

See Supplementary File 2

Supplementary Table 3: Gene expression analysis of the identified proteins in OSCC tissues using Oncomine 4.5 Research Edition

See Supplementary File 3

Supplementary Table 4: List of proteins whose genes are dysregulated in OSCC tissues as compared with noncancerous tissues

See Supplementary File 4
